# Clinical and laboratory characteristics of systemic anaplastic large cell lymphoma in Chinese patients

**DOI:** 10.1186/1756-8722-5-38

**Published:** 2012-07-07

**Authors:** Yan-Fang Wang, Yan-Li Yang, Zi-Fen Gao, Chun-Ju Zhou, Xylina Gregg, Yun-Fei Shi, Jing Wang, Xiao-Feng Yang, Xiao-Yan Ke

**Affiliations:** 1 Department of Hematology and Lymphoma Research Center, Peking University, Third Hospital, Beijing, P. R, 100191, China; 2 Department of Pathology, Peking University Health Science Center, Beijing, P. R., 100191, China; 3 Department of Pathology, Beijing Children’s Hospital, Beijing, P. R., 100045, China; 4 Utah Cancer Specialists, Salt Lake City, UT, 84106, U.S.A; 5 Department of Pharmacology, Temple University School of Medicine, Philadelphia, PA, 19140, U.S.A

**Keywords:** Systemic anaplastic large cell lymphoma, Prognosis, Anaplastic lymphoma kinase, Ki-67, BCL-2, WT1

## Abstract

**Background:**

Systemic anaplastic large cell lymphoma (S-ALCL) is a rare disease with a highly variable prognosis and no standard chemotherapy regimen. Anaplastic lymphoma kinase (ALK) has been reported as an important prognostic factor correlated with S-ALCL in many but not all studies. In our study, we retrospectively analyzed 92 patients with S-ALCL from the Peking University Lymphoma Center for clinical and molecular prognostic factors to make clear the role of ALK and other prognostic factors in Han Chinese S-ALCL.

**Results:**

The majority of Chinese S-ALCL patients were young male patients (median age 26, male/female ratio 1.7) and the median age was younger than previous reports regardless of ALK expression status. The only statistically significant different clinical characteristic in S-ALCL between ALK positive (ALK^+^) and ALK negative (ALK^-^) was age, with a younger median age of 22 for ALK^+^ compared with 30 for ALK^-^. However, when pediatric patients (≤18) were excluded, there was no age difference between ALK^+^ and ALK^-^. The groups did not differ in the proportion of males, those with clinical stage III/IV (49 vs 51%) or those with extranodal disease (53 vs 59%). Of 73 evaluable patients, the 3-year and 5-year survival rates were 60% and 47%, respectively. Univariate analysis showed that three factors: advanced stage III/IV, lack of expression of ALK, and high Ki-67 expression, were associated with treatment failure in patients with S-ALCL. However, ALK expression correlated with improved survival only in patients younger than 14 years, while not in adult patients. In multivariate analysis, only clinical stage was an independent prognostic factor for survival. Expressions of Wilms tumor 1 (WT1) and B-cell lymphoma 2 protein (BCL-2) correlated with the expression of ALK, but they did not have prognostic significance. High Ki-67 expression was also a poor prognostic factor.

**Conclusions:**

Our results show that ALK expression alone is not sufficient to determine the outcome of ALCL and other prognostic factors must be considered. Clinical stage is an independent prognostic factor. Ki-67 expression is a promising prognostic factor.

## Background

Primary anaplastic large cell lymphoma (ALCL) occurs as a systemic form (S-ALCL) and a cutaneous form, which are clinically and pathologically distinct disorders of mature T cells. S-ALCL, first described by Stein and colleagues in 1985 [1], accounts for only 2–8% of non-Hodgkin’s lymphomas in adults and 10–15% in children [2]. About 60% of S-ALCL express anaplastic lymphoma kinase (ALK), a chimeric protein with tyrosine kinase activity that is most commonly created by a unique chromosomal translocation t(2;5)(p23;q35) resulting in the fusion of the *ALK* gene with the nucleophosmin (*NPM*) gene [3,4].

S-ALCL is highly variable in morphology, phenotype, and clinical course. The prognosis of S-ALCL has been reported to correlate with the expression of ALK [5,6]. ALK^+^ S-ALCL often occurs in males younger than 30 years and is associated with a more favorable prognosis, while ALK^-^ S-ALCL usually occurs in older patients (> 60 years), affects both genders and has an unfavorable prognosis. However, different reports have reached various conclusions about the correlation of various prognostic factors with survival [6-10], and these reports also have conflicting data regarding features associated with ALK^+^ versus ALK^-^ S-ALCL. For example, Gascoyne *et al.*[6] found that ALK expression, age, performance status, lactate dehydrogenase (LDH), extranodal disease, and international prognostic index (IPI) were significant prognostic factors, while stage was not a predictor of outcome. Falini *et al.*[7] also showed that ALK^+^ and a low or low-intermediate IPI were independent variables predicting survival in multivariate analysis. One of the largest studies [8], a multinational collaboration involving 22 institutions and 159 patients with S-ALCL, reported that stage, LDH, and IPI were prognostic factors, but ALK expression was a favorable prognostic factor only in younger patients. Park *et al.*[9] reported that IPI and age were predictors of outcome, whereas ALK expression did not affect prognosis. Another retrospective study from Sibon *et al.*[10] reported that ALK expression affected prognosis only for older patients and ALK was not an independent prognostic factor.

Our previous study showed that the prevalence and clinical and laboratory characteristics of lymphomas among Han people in China may be different from that in other populations [11,12], suggesting that genetic background and environmental factors may affect the genesis, expression of biomarkers, and prognosis of lymphomas. So, it is necessary to analyze the clinical features associated with ALK^+^ and ALK^-^ in a Han Chinese patient population and their prognostic significance. Furthermore, in order to find a better combination of prognostic factors for S-ALCL, we also focused on identifying other prognostic biomarkers for S-ALCL. A number of biomarkers have been identified as prognostic factors in various hematopoietic malignancies [13-22]. Since stress-induced apoptosis is a primary pathway involved in chemotherapy-induced cell death [23,24], we set up to determine whether some apoptosis-related biomarkers have potential prognostic value in S-ALCL. B-cell lymphoma 2 (BCL-2), an anti-apoptotic gene, is expressed in several subtypes of lymphomas, wherein a high level of expression of BCL-2 has been associated with poor outcome [13-15]. The expression of Ki-67, a nuclear antigen protein, is used as a reflection of proliferation and metastatic potential of non-Hodgkin's lymphoma (NHL) [16,17]. Wilms tumor 1 (WT1) is a tumor suppressor gene and a regulator of apoptosis [18], which is expressed in various types of solid tumors and several hematologic malignancies. WT1 expression is considered an unfavorable prognostic marker in acute leukemia [19-21] and WT1 expression is also detected in S-ALCL [22]; however, the prognostic value of BCL-2, WT1, and Ki-67 in S-ALCL remains to be determined.

In this study, we retrospectively analyzed the data of 92 patients with S-ALCL from a single referral center, the Peking University Lymphoma Center, for clinical features, expression rates of ALK, BCL-2, WT1 and Ki-67, and their association with outcome to determine potential combinations of factors in predicting the prognosis of S-ALCL.

## Methods

### Patients

During a ten-year period from 2001 to 2011, 92 S-ALCL patients at the Peking University Third Hospital and the Lymphoma Laboratory of Peking University Health Science Center were enrolled in the study. Primary cutaneous anaplastic large-cell lymphoma was excluded from the study. Of the 92 cases, 38 cases were pediatric patients with age of 18 years or younger. In accordance with a protocol approved by the medical ethics committee at Peking University Health Science Center, retrospective analyses on the patients’ data were carried out. Data collected included: age, gender, Ann Arbor clinical stage, date of diagnosis, disease sites (nodal or extranodal), presence of B symptoms, and survival status. Ann Arbor stage [25] was determined by utilizing history, physical examination, chest, abdominal and pelvic computed tomography scans, and bone marrow aspirate and biopsy. If abnormal lymphocytes were found by any of the methods used, including cytomorphologic inspection of bone marrow smear, biopsy or flow cytometry, we thought that the patient have bone marrow involvement. There were not primary or acquired immunodeficiency diseases in our cases. All patients received CHOP or CHOP-like combination chemotherapy regimens. Treatment outcome was determined by overall survival (OS), which was defined as the time from diagnosis to the last contact date or to death from any cause, with 19 patients lost to follow up and unavailable for survival analyses.

### Histopathologic analysis

The S-ALCL patients were diagnosed according to the World Health Organization Classification [26]. The diagnosis of S-ALCL was based both on the histopathologic presence of large cells with anaplastic morphology (pleomorphic nuclei, prominent nucleoli, and abundant cytoplasm) and on the expression of CD30 antigen by the tumor cells. There is no clear difference in the morphological features between ALK^+^ and ALK^-^ ALCL and the criteria that divide S-ALCL into ALK^+^ and ALK^-^ subgroups is based on the expression of ALK. CD30 is also an important index to distinguish ALK^-^ ALCL from peripheral T cell lymphoma, NOS; CD15, CD45, and PAX-5 were used to exclude Hodgkin lymphoma from the study. EBV was detected by in situ hybridization of EBER. HTLV-1 is not a routine test for S-ALCL in our pathological laboratory.

### Immunohistochemical staining

Formalin-fixed, paraffin-embedded tissue samples from the 92 patients were used for further immunohistochemical analyses. BCL-2, WT1, Ki-67, and ALK in tissue sections were immuno-stained using specific antibodies followed with the diaminobenzidine (DAB) histochemistry kit (Molecular Probes, Invitrogen, California, USA) as we previously reported [12]. Briefly, 4 μm thick tissue sections from paraffin blocks were de-paraffinated and heat-retrieved for antigens in target retrieval solution (pH 6.0) in a microwave oven for 2–3 min, depending on the size of the section. Sections were then incubated with anti-BCL-2, WT1, Ki-67, and ALK antibodies (Dako-China Branch, Shanghai, China), respectively, for one hour followed by biotinylated rabbit anti-mouse antibody and horseradish peroxidase (HRP)-conjugated avidin. Finally, DAB staining on the sections were visualized under bright field light microscopy. The expressions of WT1 and BCL2 in tissues were divided into positive and negative groups using 10% as a threshold value [27]. The proliferation index was calculated as the ratio of Ki-67 positive nuclei with respect to the total number of neoplastic cells, which was used to classify tumors into two types, low proliferation (≤30% Ki-67 positive neoplastic cells) or high proliferation (>30% Ki-67 positive neoplastic cells) [28] .

### Statistical analysis

Estimates of OS and univariate analyses of prognostic factor were performed by using the Kaplan-Meier method. The positive rates of biomarkers in S-ALCL were analyzed by using the Chi-square test. Multivariate analyses were performed with the Cox proportional hazard regression model (enter and remove limits 0.1). *P* values below 0.05 were considered to be significant. All the analyses were performed using the SPSS statistical software package (version 13.0, SPSS, Chicago, USA).

## Results

### Gender, age, and stage composition of Chinese S-ALCL patients

Of the 92 patients with S-ALCL, 58 were male and 34 were female, for a male/female ratio of 1.7. The median age was 26 years (range 2 to 74) and 38 patients (41%) were 18 years or younger. Clinical staging analysis showed that 50% patients were stage III to IV and 47% patients had systemic B symptoms, predominantly fever. Forty-one patients (45%) had nodal-only disease, but the majority (55%) had extranodal tissue involvement, including lung, bone marrow, liver, spleen, and skin.

### Histopathology and immunophenotype

Most of the cases had the typical pleomorphic cytology and sinusoidal infiltration. So-called hallmark cells with eccentric horse-shoe or kidney-shaped nuclei were present both in ALK^+^ or ALK^-^ cases. Five cases (5%) of small-cell variant of ALCL were included in the study. 61 cases (66%) were common “T cell” phenotype with the expression of CD3 and/or CD43, and CD45RO. 31 cases (34%) had an apparent “null cell” phenotype. EBV was consistently negative in all S-ALCL cases.

### Clinical features stratified by expression of ALK

The clinical features stratified by expression of ALK are shown in Table 1. Patients with ALK^+^ S-ALCL were significantly younger than those with ALK^-^ S-ALCL (median age 22 vs. 30 years) (*P*<0.05), but both subgroups had a predominance of males, and a similar proportion of stage III/IV disease (ALK^+^ 49%; ALK^-^ 51%). B symptoms were present in 55% of ALK^+^ and in 37% of ALK^-^ patients, but this did not reach statistical significance. Extranodal involvement was similarity in ALK^+^ vs. ALK^-^ (53 vs. 59%). Although not statistically significant, the most common sites of involvement in ALK^+^ S-ALCL were subcutaneous tissue (14%), lung (14%), skin (10%), and bone (6%), and in ALK^-^ S-ALCL were skin (20%), spleen (15%), lung (10%) and bone (10%). The frequency of bone marrow involvement was lower in ALK^+^ S-ALCL (0% vs. 5% in ALK^-^ ALCL), but this was also not statistically significant (Table 2).

**Table 1:** Clinical characteristic of S-ALCL patients, according to ALK expression

**Clinical feature**	**ALK**^**+**^**S-ALCL**	**ALK**^**-**^**S-ALCL**	**P**
**patients no.**	**51**	**41**	
**Median age (all patients) (range), y**	**22(3–63)**	**30(2–74)**	**<0.05**
**Median age (without pediatric patients) (range), y**	**38(19–63)**	**34(19–74)**	
**Male–female ratio**	**1.7:1**	**1.7:1**	**1.000**
**Stage, no.(%)**			**1.000**
**I**	**16(31)**	**11(27)**	
**II**	**10(20)**	**9(22)**	
**III**	**17(33)**	**13(32)**	
**IV**	**8(16)**	**8(19)**	
**location of tumor, no. (%)**			**0.675**
**nodal**	**24(47)**	**17(41)**	
**extranodal**	**27(53)**	**24(59)**	
**B symptom, no. (%)**			**0.095**
**with**	**28(55)**	**15(37)**	
**without**	**23(45)**	**26(63)**	

**Table 2:** **Extranodal sites of involvement in ALK**^**+**^**and ALK**^**-**^**S-ALCL**

**Extranodal sites**	**ALK**^**+**^**S-ALCL(%)**	**ALK**^**-**^**S-ALCL(%)**	**P**
**Bone marrrow**	**0**	**2(5)**	**0.196**
**Subcutaneous tissue**	**7(14)**	**3(7)**	**0.503**
**Lung**	**7(14)**	**4(10)**	**0.749**
**Bone**	**3(6)**	**4(10)**	**0.696**
**Skin**	**5(10)**	**8(20)**	**0.234**
**Liver**	**2(4)**	**3(7)**	**0.653**
**Spleen**	**2(4)**	**6(15)**	**0.133**
**Epidural**	**0**	**1(2)**	**0.446**
**Pleural effusion**	**2(4)**	**2(5)**	**1**
**Pericardial effusion**	**0**	**2(5)**	**0.196**

Furthermore,54 patients aged 19 years or older were analyzed separately, with the following findings (Table 3). The median age was 38 years in the ALK^+^ S-ALCL group and 34 years in the ALK^-^ S-ALCL group. B symptoms were present in 39% of ALK^+^ and in 35% of ALK^-^ patients. Forty-three percent of ALK^+^ patients had stage III/IV disease versus 52% of ALK^-^. The most common extranodal sites in this patient population were skin (10%), lung (8%), and bone (6%) for ALK^+^ patients, and skin (16%), spleen (13%), liver (6%), and pleural effusion (6%) for ALK^-^ patients.

**Table 3:** Clinical features of Chinese S-ALCL compared with Savage’s report

**Clinical feature**	**ALK**^**+**^**S-ALCL**		**P**	**ALK**^**-**^**S-ALCL**		**P**
	**This report**	**Savage*et al.***		**This report**	**Savage*et al.***	
**Total no.patients (≥19 years)**	**23**	**87**		**31**	**72**	
**Median age, y**	**38**	**34**		**34**	**58**	
**Age less than 60 y, no. (%)**	**22(96)**	**74(86)**	**0.175**	**26(84)**	**42(58)**	**<0.05**
**Male–female ratio**	**2.8:1**	**1.7:1**	**0.34**	**1.8:1**	**1.5:1**	**0.68**
**Stage, no.(%)**			**0.07**			**0.53**
**I**	**9(40)**	**1(0)**		**8(26)**	**0(0)**	
**II**	**4(17)**	**30(35)**		**7(23)**	**30(42)**	
**III**	**6(26)**	**25(29)**		**10(32)**	**15(21)**	
**IV**	**4(17)**	**31(36)**		**6(20)**	**27(37)**	
**Nodal-only disease, no. (%)**	**11(48)**	**39(54)**	**0.8**	**13(42)**	**38(49)**	**0.31**
**Extranodal sites more than 1, no. (%)**	**4(17)**	**17(19.5)**	**0.82**	**5(16)**	**15(21)**	**0.58**
**Extranodal sites, no. (%)**						
**Bone marrrow**	**0**	**10(12)**	**0.09**	**2(5)**	**5(7)**	**0.93**
**Subcutaneous tissue**	**0(0)**	**9(10)**	**0.1**	**1(3)**	**2(3)**	**0.9**
**Lung**	**2(8)**	**7(8)**	**0.92**	**1(3)**	**9(13)**	**0.14**
**Bone**	**3(6)**	**12(14)**	**0.93**	**2(5)**	**5(7)**	**0.93**
**Skin**	**5(10)**	**7(8)**	**0.06**	**5(16)**	**12(17)**	**0.95**
**Liver**	**1(4)**	**3(3)**	**0.84**	**2(6)**	**7(10)**	**0.59**
**Spleen**	**1(4)**	**9(10)**	**0.37**	**4(13)**	**2(3)**	**<0.05**
**Pleural effusion**	**1(4)**	**3(3)**	**0.84**	**2(6)**	**4(6)**	**0.86**
**Pericardial effusiom**	**0(0)**	**0(0)**	**0.6**	**1(3)**	**1(1)**	**0.54**
**B symptoms, no. (%)**	**9(39)**	**52(60)**	**0.08**	**11(35)**	**41(57)**	**<0.05**
**5-y OS,** %	**58**	**70**		**35**	**49**	

### Achievement of CR is associated with a longer median survival time

**Figure 1:**
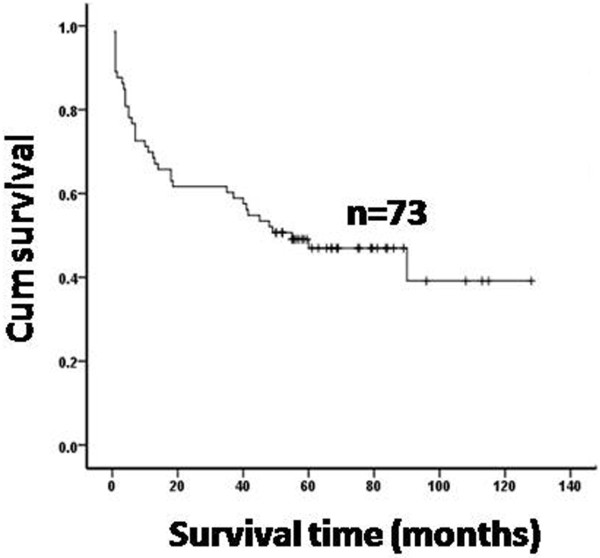
**Overall survival (OS) of the 73 evaluable patients with S-ALCL**. The median survival time is 67 months and the 5-year OS rate is 47%. n = total number of the evaluable patients with S-ALCL.

Of the 73 evaluable patients, the median time of follow-up was 67 months (range 0.5 months to 128 months). We determined the overall survival (OS) of patients using the Kaplan-Meier analytic method. The mean survival time of the 73 evaluable patients was 67 months (95% confidence intervals: 53–80 months) and the 3- and 5-year OS rates were 60% and 47%, respectively (Figure 1). Among the 34 surviving patients, 26 (76%) patients had achieved a complete remission (CR) after induction chemotherapy, 7 (21%) had a partial remission (PR), and 1 (3%) patients was primary refractory. The median survival time of the patients with a CR was significantly longer than others (77 months vs.59 months, P<0.05). Of the 39 deceased patients, 22 (56%) died in the first year after diagnosis and 28 (72%) had stage III–IV disease.

### Stage is an independent prognostic factor for survival

**Figure 2:**
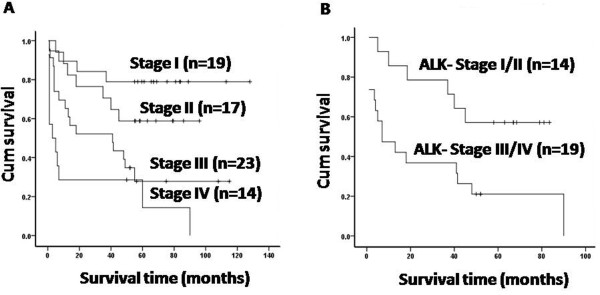
**Overall survival by Ann Arbor stage**. (A) The 5-year OS of 73 evaluable patients with S-ALCL by stage I, II, III and IV is 78%, 59%, 28% and 14%, respectively (*P = 0.000*). (B) The 5-year OS of 33 patients with ALK^-^ S-ALCL by stage I/II and III/IV is 58% and 20%, respectively (*P = 0.016*). n = patient numbers in each group.

The following clinical variables were evaluated for the 73 evaluable patients to determine their utility in predicting prognosis: age, gender, stage, extranodal involvement, and B symptoms. However, only the clinical stage significantly correlated with prognosis in univariate analysis. The 5-year OS rates for patients with stage I, II, III, IV were 78%, 59%, 28%, and 14%, respectively (*P*<0.05) (Figure 2A). Furthermore, the prognostic value of stage could be seen in ALK^-^ S-ALCL, while not in ALK^+^ S-ALCL. In the 33 ALK^-^ cases, the 5-year OS rate for stage I/II was 58% and for stage III/IV was 20% (*P*<0.05) (Figure 2B), which suggest that ALK^-^ S-ALCL patients could be further divided into different risk groups according to clinical staging. In addition, stage correlated with prognosis both for pediatric patients (*P*<0.05) and adult patients (*P*<0.05), suggesting that stage is not affected by age. Furthermore, we found that the clinical stage remained a significant prognostic factor in multivariate analysis (*P*<0.05).

### ALK expression is associated with a better OS, but only in patients 14 years old or younger

**Figure 3:**
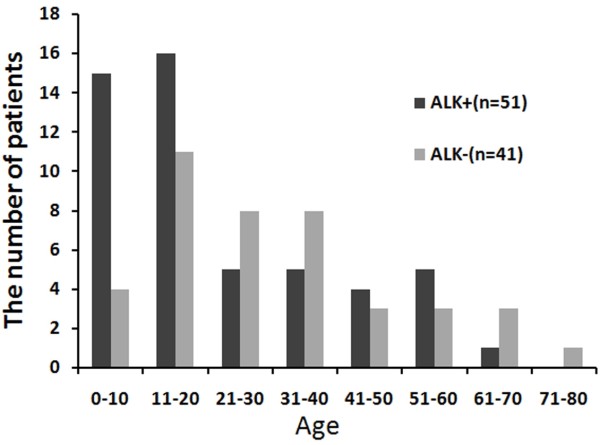
**Distribution of ALK**^**+**^**and ALK**^**-**^**S-ALCL patients by age groups.** n = total patient numbers in each group.

**Figure 4:**
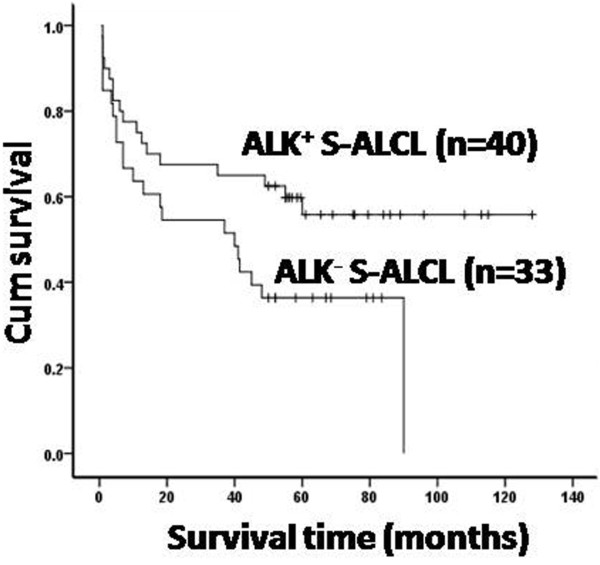
**Overall survival of the 73 evaluable patients with S-ALCL by expression of ALK**. The 5-year OS of ALK^+^ S-ALCL and ALK^-^ S-ALCL is 58% and 36%, respectively (*P = 0.044*). n = patient numbers in each group.

Fifty-five percent of the 92 cases were ALK positive. As shown in Figure 3, the rate of ALK expression was significantly higher in pediatric patients compared with adult patients (76% of those ≤18 years old compared with 42% of those >18 years (*P*<0.05)). Using Kaplan-Meier analysis, we examined the influence of ALK on the OS and found that patients with ALK^+^ S-ALCL had a more favorable prognosis than patients with ALK^-^ S-ALCL (*P*<0.05). The 5-year OS rates in the ALK^+^ group and the ALK^-^ group were 58% and 36%, respectively (Figure 4). Surprisingly, the prognostic value of ALK in pediatric and adult patients was different. The favorable prognosis of ALK^+^ S-ALCL was seen in the population ≤14 years old (*P* = 0.05), but not in those older than 14 years. However, in multivariate analysis, when other clinical factors and biomarkers were added, ALK was no longer a prognostic factor, regardless of age.

### High expression of Ki-67 is associated with a poorer prognosis

The expression of Ki-67 was analyzed in 74 of 92 S-ALCL cases and the results for the ALK^+^ and ALK^-^ groups are shown in Table 4. The median expression of Ki-67 in lymphoma cells was 71% (5–95%) and Ki-67’s expression was not statistically correlated with the expression of ALK, with a median expression of Ki-67 in the ALK^+^ and ALK^-^ group of 60% and 80%, respectively. Seventy-four percent of S-ALCL had high-expression (>30%) of Ki-67 and 26% had low-expression (≤30%) (Table 4), suggesting that most S-ALCL has a high proliferating status.

**Table 4:** Relation of expression between Ki-67, BCL-2, WT1 and ALK

**Index**	**ALK**		**total**	***χ2***	**P**
	**(+)**	**(−)**	**no.(%)**		
**Ki-67**				**0.268**	**>0.05**
**low-expression, no.**	**12**	**7**	**19(26)**		
**high-expression, no.**	**31**	**24**	**55(74)**		
**total, no.**	**43**	**32**	**74(100)**		
**BCL-2**				**5.336**	**<0.05**
**(+), no.**	**4**	**7**	**11(19)**		
**(−), no.**	**35**	**13**	**48(81)**		
**total, no.**	**39**	**20**	**59(100)**		
**WT1**				**4.479**	**<0.05**
**(+), no.**	**27**	**8**	**35(61)**		
**(−), no.**	**11**	**11**	**22(39)**		
**total, no.**	**38**	**19**	**57(100)**		

**Figure 5:**
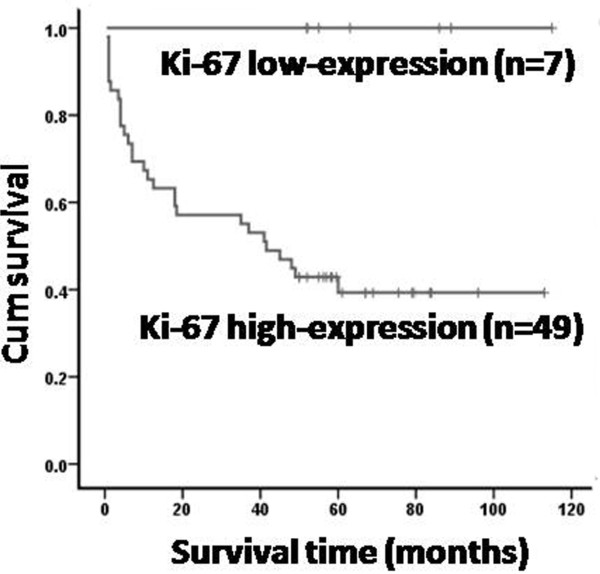
**Overall survival stratified by Ki-67 expression.** The 5-year OS of ki-67 low expression S-ALCL and ki-67 high expression S-ALCL is 100% and 39%, respectively (*P = 0.013*). n = patient numbers in each group.

On further evaluation of 56 patients, we found that patients with Ki-67 low-expression had a better outcome compared with those with Ki-67 high-expression, with a 5-year OS of 100% versus 39% (*P*<0.05), (Figure 5). When the analysis was limited to the ALK^+^ or ALK^-^ S-ALCL patients, Ki-67 high-expression was not a prognostic factor. However, we found a prognostic value of Ki-67 in pediatric patients (*P*<0.05), but not in adult patients, suggesting that the role of Ki-67 could be affected by age. Further, in multivariate analysis, Ki-67 high-expression is no longer correlated with poorer prognosis.

### BCL-2 expression is inversely correlated with ALK expression but is not significantly associated with prognosis

BCL-2 expression in tumor cells was examined in 59 cases. Overall, 11 (19%) patients were BCL-2 positive. No differences were observed between the BCL-2-positive group and BCL-2-negative group in gender, age, nodal involvement, and clinical stage. The expression of BCL-2 was significantly inversely associated with the expression of ALK, with BCL-2 positivity in 10% (4 of 39) of the ALK^+^ group and 35% (7 of 20) of the ALK^-^ group (*P*<0.05) (Table 4).

Although previous studies reported that BCL-2 expression is related to poor clinical outcome in several lymphomas [13,14], we did not find any prognostic value of BCL-2 expression in patients with S-ALCL in neither univariate analysis nor in multivariate analysis.

### WT1 expression is positively correlated with ALK expression but is not significantly associated with prognosis

To address if WT1 expression is a prognostic factor in S-ALCL, we performed immuno- histochemical staining of WT1 in 57 cases and found positive expression of WT1 in 35 (61%) cases. Table 4 summarizes the expression of WT1 in the ALK^+^ group and the ALK^-^ group. WT1 expression positively correlated with ALK expression. WT1 positive rates were 71% (27 of 38)and 42% (8 of 19)in the ALK^+^ group and the ALK^-^ group, respectively (*P* < 0.05). However, WT1 over-expression was not a prognostic factor for patients with S-ALCL neither in univariate analysis nor in multivariate analysis.

## Discussion

S-ALCL is a lymphoma with an aggressive and heterogeneous clinical course, primarily occurring in children and young adults. Most investigators reported that the response of ALCL to chemotherapy was good, especially in children, ranging from 60%–90% [2,9,29]. However, for patients at high-risk for treatment failure, other therapies such as high-dose chemotherapy followed by stem cell therapy may be needed to improve the long-term survival [30]. Therefore, identification of prognostic factors would aid clinicians in selecting an optimal therapeutic regimen.

In this study, we analyzed clinical characteristics and prognostic factors in Han Chinese patients with S-ALCL from a single referral center. We found some unique clinical features in our S-ALCL patients. The median age of our patients was 26 years regardless of ALK expression status, and even when pediatric patients were excluded, the median age of ALK^-^ S-ALCL was 34, which is much younger than other reports [6-10,30-32]. The percentage of patients with clinical stages III/IV in our study was also less than previous reports (50 vs. 56%–72%) [6-10,30-32]. Since some studies [8,33] excluded pediatric patients, we also analyzed the subset of patients aged 19 years or older and compared the results to those reported by *Savage et al.*[8]. We found that some of the clinical features of ALK^+^ S-ALCL in our analysis were similar to those in *Savage et al.*, but Chinese patients with ALK^-^ S-ALCL are younger (median age 34 vs. 58), with a lower percentage of B symptoms (35 vs. 57%), and different sites and frequency of extranodal involvement, compared to those reported by *Savage et al.*[8]*.* Our study found that skin, spleen, lung, and bone were common sites in ALK^-^ S-ALCL, while in Savage’s report bone marrow, subcutaneous tissue, bone, liver, and spleen were often involved. The 5 year-OS of our S-ALCL patients was also lower than Savage’s report; however, their patient population had varied ethnic backgrounds (25% North American, 24% European, and 40% Asian), which might account for some of these differences.

Our study found that patients who had a CR following induction chemotherapy had a more favorable prognosis, suggesting that achieving a CR during induction chemotherapy is a principal factor of longer OS. In our cohort of patients, most deaths occurred in the first year after diagnosis, which indicates that S-ALCL is a rapidly fatal disease and effective induction chemotherapy is essential for long-term survival.

Although previous studies [6,7] identified several clinical characteristics, such as advanced clinical stage, older age, extranodal involvement, and B symptoms with a high-risk disease, clinical stage was the only independent clinical prognostic marker in our study. None of the other clinical factors had statistical significance whether by univariate analysis or by multivariate analysis, consistent with the report by Savage *et al.*[8]. In addition to the clinical characteristics, we also analyzed the expression of several tumor biomarkers. We found for the first time that Ki-67 had prognostic significance in S-ALCL. Furthermore, the expression of Ki-67 also defined different risk categories in patients with ALK^-^ S-ALCL, although not in ALK^+^ S-ALCL.

ALK^+^ S-ALCL may have a more favorable clinical course than ALK^-^ S-ALCL [6-8], but recent study found that activation of ALK could provides oncogenic addiction to tumors harboring activating mutation or translocation of ALK such as in non-small cell lung cancer, so ALK inhibitor may be a potent novel targeted therapeutic in some solid tumor [34]. In our Han Chinese patients, the 5-year OS was 58% in ALK^+^ S-ALCL patients versus 36% in ALK^-^ S-ALCL patients, which was lower than other reports showing 70%-80% survival in ALK^+^ versus 33%-49% in ALK^-^ S-ALCL [6,7,31,32]. Though poorer outcome for ALK^-^ S-ALCL patients, they may be divided into different groups by stage or expression of Ki-67. Recently, *Savage et al.*[8] found that the favorable outcome of ALK^+^ S-ALCL was restricted to younger patients and no outcome differences were seen between the ALK^+^ and ALK^-^ groups in patients over 40 years old. In contrast, *Sibon et al.*[10] reported that there was no impact of ALK status on survival in patients younger than 40 years old and *Park et al.*[9] also reported no predictive value for ALK expression. *Wang et al.*[35] thought that suppressor Tregs in ALK^+^ S-ALCL could suppress the anti-tumor immune response induced by effector T cells, which maybe weaken the role of ALK. Besides, *Beltran et al.*[36] found the favorable prognostic role of ALK expression in DLBCL and the clinical course of ALK^-^ DLBCL was aggressive. Compared to the previous reports, in our study, the favorable prognostic influence of ALK expression seems to be confined to pediatric patients. In univariate analysis, in patients who are 14 years old or younger, the ALK^+^ group had better survival rate than ALK^-^ group, but this survival difference was not present in patients older than 14 years. Nevertheless, in multivariate analysis, ALK was no longer a significant prognostic factor whether in pediatric or adult patients. These results suggest that the prognostic value of ALK expression may be affected by other as yet unknown factors. Clinical stage or biomarkers may ultimately be more important predictors of prognosis.

It is not known why some patients with ALK^+^ S-ALCL have better outcomes. One study showed that the tumor cells of ALK^+^ S-ALCL could be associated with higher levels of apoptosis by chemotherapeutic drugs than those of ALK^-^ S-ALCL [37]. Stress-induced apoptosis is a pathway involved in chemotherapy-induced cell death, and BCL-2 may inhibit the cell death pathway by suppressing the function of pro-apoptotic molecules [23,24]. Previous studies found that BCL-2 expression is almost completely restricted to ALK^-^ S-ALCL and is correlated with a poor outcome [13,14], however, in our study, BCL-2 expression did not have a significant influence on long-term survival, although the expression of BCL-2 was more frequently found in ALK^-^ patients than in ALK^+^ S-ALCL patients. Nevertheless, to fully evaluate the role of BCL-2 in S-ALCL, a larger number of samples would be needed.

The role of WT1 as an unfavorable prognostic marker has been confirmed in acute leukemia and some of solid tumors [20,21]. Unlike its role in acute leukemia, WT1 was not a significant prognostic factor in S-ALCL in our study. Over-expression of WT1 was more frequently found in ALK^+^ S-ALCL and there was a significant positive correlation between the expression of WT1 and ALK. This may indicate that ALK expression is affected by multiple factors and may explain why ALK was no longer a prognostic marker in our multivariate analysis. The functional interaction of ALK and WT1 in S-ALCL is a worthwhile target for future studies, although larger studies are required to validate the prognostic value of WT1 expression in S-ALCL.

In conclusion, the Han Chinese patients with S-ALCL in our study had some unique clinical features, including younger age distribution and a slightly higher percentage of early stage. Our data add to other reports stressing a potential contribution of ethnic and racial background on clinical and biological characteristics of hematological malignancies. Similar to other reports, in our study the expression of ALK and Ki-67 and clinical stage are significant prognostic factors for S-ALCL patients in univariate analysis. However, clinical stage is the only independent prognostic marker in multivariate analysis. Nevertheless, it is possible that these prognostic factors may play a role in different age groups and different populations. A larger scale and preferably multi-institutional study will be needed to confirm the prognostic role of geographical or ethnic differences. Further characterization of these and other prognostic factors in S-ALCL patients will provide better prognostic guidance for stratifying patients for future therapeutic trials.

## Competing interests

The authors declare no potential conflict of interest.

## Authors’ contributions

XY.K. designed the research and analyzed data and wrote the paper. YF.W. collected data, analyzed data and wrote the paper. YL.Y. performed research and collected data. ZF.G., CJ.Z., and YF.S. provided the pathologic sample. X.T.G. critically reviewed the data and the literature and rewrote the paper. XF.Y. reviewed and corrected the paper. J.W. participated in the statistical analysis. All authors read and approved the final manuscript.
